# Impaired T-cell proliferation among HAART-treated adults with suboptimal CD4 recovery in an African cohort

**DOI:** 10.1186/1471-2172-14-26

**Published:** 2013-06-20

**Authors:** Damalie Nakanjako, Isaac Ssewanyana, Rose Nabatanzi, Agnes Kiragga, Moses R Kamya, Huyen Cao, Harriet Mayanja-Kizza

**Affiliations:** 1 Makerere University College of Health Sciences, P.O. Box 7072, Kampala, Uganda; 2 Infectious Diseases Institute, Makerere University College of Health Sciences, Kampala, Uganda; 3 Joint Clinical Research Center, Kampala, Uganda; 4 California Department of Public Health, Richmond, CA, USA

**Keywords:** T-cell proliferation, Immune activation, Suboptimal immune recovery, HAART immune responses, HIV/AIDS

## Abstract

**Background:**

Most HIV-infected subjects exhibit a progressive rise in CD4 T-cell counts after initiation of highly active antiretroviral therapy (HAART). However, a subset of individuals exhibit very poor CD4 T-cell recovery despite effective control of HIV-RNA viraemia. We evaluated CD4 T-cell proliferation among suboptimal responders and its correlation with CD4 T-cell activation.

**Methods:**

The magnitude of CD4 increase (difference between absolute CD4 counts at baseline and absolute CD4 counts at 4 years of ART) was grouped into 4 quartiles for the 211 patients with sustained HIV-RNA viral suppression. Cases of **‘Suboptimal immune responders’** included patients within the lowest quartile [Median CD4 increase 165 (Range −43-298) cells/μl; n=52] and a comparison group of **‘Optimal immune responders’** was defined as patients within the highest quartile of CD4 increase [Median CD4 increase 528 (Range 417–878) cells/μl; n=52]. Frozen PBMC were thawed and analysed from a convenient sample of 39 suboptimal responders and 48 optimal responders after 4 years of suppressive antiretroviral therapy. T-cell activation was measured by proportions of T-cells expressing surface marker CD38 and HLADR (CD4+CD38+HLA-DR+ and CD8+CD38+HLA-DR+ cells). T-cell proliferation was determined by the extent of carboxyfluorescein diacetate succinimidyl ester (CFSE) dye dilution on culture day 5 of PBMCs in the presence of antigen (SEB, PPD, CMVpp65, GagA and GagD). Samples were analyzed on a FACS Calibur flow cytometer and flow data was analyzed using FlowJo and GraphPad.

**Results:**

Overall, CD4 T-cell proliferation on stimulation with SEB, PPD, CMVpp65, Gag A and Gag D.antigens, was lower among suboptimal than optimal responders; this was significant for SEB (CD4+ p=0.003; CD8+ p=0.048) and PPD antigens (CD8+ p=0.038). Among suboptimal responders, T-cell proliferation decreased with increasing immune activation (Negative correlation; slope = −0.13±−0.11) but not among optimal responders.

**Conclusion:**

T-cell immune activation and exhaustion were associated with poor proliferation among suboptimal responders to HAART despite sustained viral suppression. We recommend studies to further understand the mechanisms leading to impaired T-cell function among suboptimal responders as well as the potential role of immune modulation in optimizing CD4 count and functional recovery after HAART.

## Background

CD4 T-helper function remains critical to effective immune responses to common infections among HIV-infected individuals**.** With chronic HIV infection, CD4 T-cell function decreases with HIV-RNA viraemia [1,2]. Evidence suggests that the HIV-associated immune dysfunction is reversible with control of HIV-viraemia [2,3]. Strong HIV-specific immune responses have been observed among individuals receiving potent highly active antiretroviral therapy (HAART) for acute HIV infection [1]. Most HIV-infected subjects exhibit a progressive rise in CD4 T-cell counts after initiation of HAART [4]. However, up to 40% of HAART-treated individuals exhibit very poor CD4 T-cell recovery despite effective control of HIV-RNA viraemia [5,6]. CD4 count measurement is the main laboratory tool for monitoring immune recovery in many HIV treatment programs in sub-Saharan Africa (SSA) [7]**.** With the increasing number of individuals on HAART for longer period of time and with the emerging population of suboptimal responders to HAART despite viral suppression, there is need to consider CD4 T-cell function recovery as the ultimate measure of immune recovery. In the developed world, significant proliferative responses were observed in 30-69% of individuals on suppressive HAART [8-11]. There is paucity of data on CD4 T-cell function recovery within HIV treatment programs in SSA. Moreover, persistently low T-cell function is likely to contribute to the increased risk of opportunistic infection observed among the individuals with suboptimal CD4 reconstitution despite suppressive HAART [12]. This study compared T-cell proliferation among suboptimal and optimal responders after four years of suppressive HAART upon *in-vitro* stimulation with common antigens like Staphylococcus Enterotoxin B, Cytomegalovirus and the M. Tuberculosis antigen purified protein derive (PPD). We also determined the correlation between T-cell proliferation and immune activation among suboptimal responders to HAART. Our results give insight on the persistent immune dysfunction among patients that do not reconstitute their CD4 counts despite long periods of HIV-RNA viral suppression and emphasize the need for innovative immune modulation interventions to optimise immune recovery in this sub-population of HAART-treated individuals.

## Methods

### Study setting and participants

Between April, 2004 and April, 2005, 559 consecutive ART-naïve HIV-infected patients, were initiated on HAART and enrolled into the Infectious Diseases Institute (IDI) prospective observational research cohort as previously described [13]. Patients were initiated on first-line HAART at CD4 counts ≤ 200 cells/μl according to Ugandan guidelines for HAART initiation at the time. Drugs were provided through the Global Fund (a generic combined formulation of stavudine [d4T], lamivudine [3TC], and nevirapine [NVP] and the US President’s Emergency Plan for AIDS Relief ( a combined formulation of zidovudine [ZDV] and 3TC plus efavirenz [EFZ] or NVP). Patients with toxicity to ZDV were changed to tenofovir [TDF]. All patients received cotrimoxazole (or dapsone) prophylaxis according to the national policy to provide cotrimoxazole to all people living with HIV (PLHIV). Adherence to HAART was encouraged by at least 3 individual and group counseling sessions. Patients were reviewed monthly by the study physicians that evaluated among others, adherence to medication, toxicities and acute infections. HIV RNA viral loads, complete blood counts and CD4 lymphocyte counts were measured 6 monthly.

### Definition of suboptimal CD4 reconstitution

**Figure 1:**
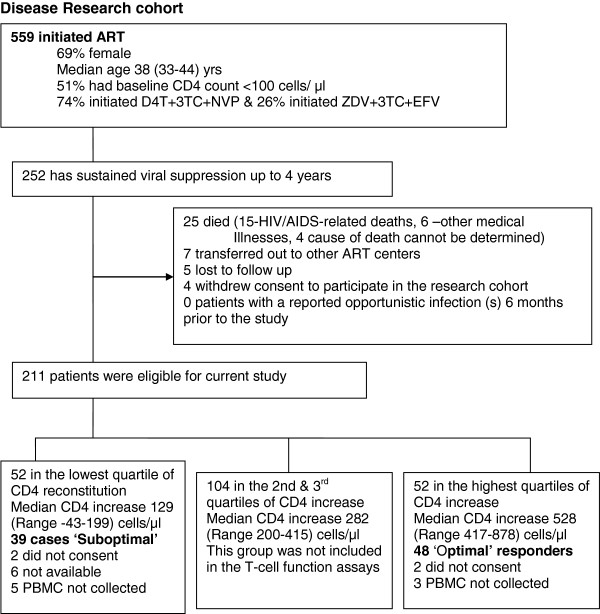
Profile of patients on antiretroviral therapy within the Infectious Diseases Institute Research cohort.

Various definitions have been used to describe suboptimal CD4 reconstitution following HAART including the magnitude of the CD4 cell increase [14]**.** Kaufmann et al. defined suboptimal CD4 reconstitution an absolute CD4 count < 500 cells/ μl after 5 years of sustained viral loads < 1000 copies [5]. The authors thought the latter criteria would over–estimate suboptimal immune response in our cohort since less than one-third of all our patients had attained an absolute CD4 count ≥ 500 cells/ μl. Therefore we used a cohort-specific definition of suboptimal CD4 reconstitution in order to consider the whole spectrum of CD4 recovery. The magnitude of CD4 increase (difference between absolute CD4 counts at baseline and absolute CD4 counts after 4 years of HAART), for the 211 patients with sustained HIV-RNA viral suppression, was grouped into 4 quartiles. Cases of **‘Suboptimal immune responders’** included patients within the lowest quartile [Median CD4 increase 165 (Range −43-298) cells/μl; n=52] and the comparison group of **‘Optimal immune responders’** included patients within the highest quartile of CD4 increase [Median CD4 increase 528 (Range 417–878) cells/μl; n=52]. This study compared CD4 T-cell proliferation among suboptimal and optimal responders. The second and third quartiles, including 104 individuals with a median CD4 increase 282 (Range 200–415) cells/μl were considered as average immune responders and were not included in this study which looked at the two extremes of immune recovery. Frozen PBMCs were from a convenient sample of 39 suboptimal responders and 48 optimal responders after 4 years of suppressive HAART (see Figure 1).

We excluded patients that had an opportunistic infection in the previous 6 months. Rapid tests for malaria antigens, stool microscopy for intestinal helminths and a C-reactive protein were done to exclude common causes of immune activation in our setting. Written informed consent was obtained from all participants. This study was approved by national review research and ethics board; the Uganda National Council for Science and Technology. The immunology assays were performed at the Cytotoxic T-lymphocyte laboratory (CTL) at the Joint Clinical Research Center (JCRC), Kampala, Uganda.

### Peripheral Blood Mononuclear Cells (PBMC) separation

Fifty (50) mls of whole blood, collected in ACD-A bottles, was processed for PBMC separation within 4 hours of collection. PBMCs were separated by Ficoll-Hypaque density configuration, washed and re-suspended in phosphate buffer saline (PBS) containing heat inactivated fetal calf serum (FCS). PBMCs were frozen and stored in Fetal Calf Serum with 10% dimethyl sulfoxide (DMSO), in liquid nitrogen until assay time. On day 0, cell surface staining was done with the following antibodies; CD3 APC, CD4 PerCP-Cy5.5, HLA-DR FITC and CD38 PE for immune activation and PD-1 APC for immune exhaustion (BD Biosciences San Jose, CA). Samples were analyzed on a FACS Calibur flow cytometer (BD Biosciences, San Jose, CA). Overall, at least 50,000 events in the CD3-positive gate were collected. The gating was standardised and set using fluorescence minus one controls for HLADR and CD38.

### T-cell proliferation assays

**Figure 2:**
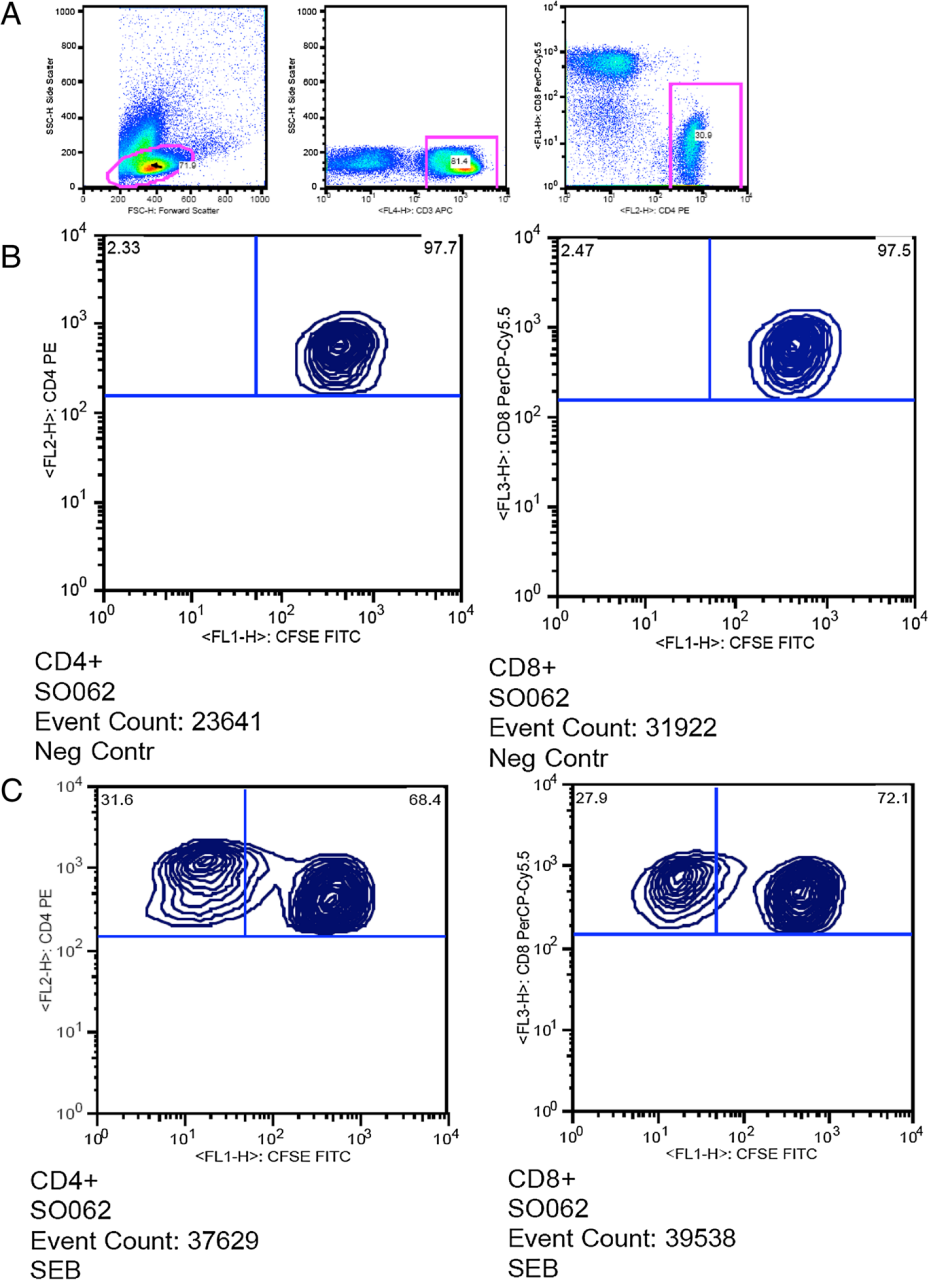
**Gating strategy for T-cell proliferation using CFSE dye and SEB antigen stimulation.** Panel **A** shows the gating for CD T-lymphocytes, Panel **B** shows the negative control without CD4 and CD8 T-cell proliferation and Panel **C** shows an individual displaying CD4 and CD8 proliferation upon stimulation with SEB.

Cell proliferation was determined by carboxyfluorescein diacetate succinimidyl ester (CFSE) dilution using the CellTrace™ CFSE Cell Proliferation Kit (Invitrogen, Carlsbad, CA), per the manufacturer’s instructions. Previously thawed PBMC, which had been rested overnight were cultured in the presence of either, Staphylococcal enterotoxin B (SEB) Sigma-Aldrich, St,Lois, MO, Purified Protein Derivative (PPD), Q Biogene Carlsbad, CA, USA, CMVpp65- μL (Q Biogene Carlsbad, CA, USA), Gag A and Gag D (NIH/NIAID repository) or un stimulated at 37°C and 5% CO_2,_ for five days. The final concentration of the antigens was 1μg/ml of PBMC (1 million PBMC per well), as previously optimised by our team [15]. On day 5, the cells were harvested and stained with CD3 APC, CD4 PE, CD8 PerCp-Cy5.5 (BD Biosciences, San Jose, CA). The flowcytometry data analysis was performed using FlowJo software (TreeStar, Version X). Only data with a minimum of 10,000 acquired events of CD3+CD4+ or CD3+CD8+ were analyzed. T-cell proliferation was determined by the extent of carboxyfluorescein diacetate succinimidyl ester (CFSE) dye dilution on culture day 5 of thawed PBMCs in the presence of antigen (PPD, CMVpp65, GagA and GagD). Staphylococcal Enterotoxin B (SEB Sigma-Aldrich, St. Louis, MO) stimulation was used as a positive control. All the study participants demonstrated significant proliferation following SEB stimulation. Proliferation results ≤ background response (un stimulated PBMC) were considered negative whereas results ≥ twice the background values (after subtraction of background) were considered positive. Figure 2 shows the gating strategy we used to analyse T-cell proliferation using CFSE dye and SEB antigen stimulation.

### Statistical analysis

Using Prism Graph Pad 5.0 software, we compared proportions of proliferated CD4+ and CD8+ T-cells among optimal and suboptimal responders; using the Mann Whitney test for non-parametric variables. *P* values <0.05 were considered statistically significant. We also determined the linear correlation between T-cell proliferation and immune activation (co-expression of CD38 and HLADR cell surface markers).

## Results

### Study participants

After 4 years of follow up on HAART, 252/559 (45%) patients had sustained HIV-RNA viral suppression. Of these, 41 were excluded due to the following reasons; death (n=25), lost to follow-up (n=5), voluntary request to transfer to and voluntary termination from the study (n=11). The median age [Inter Quartile Range (IQR)], was 36 (31–42) years, Body Mass Index (BMI); 22 (IQR 20–72), and haemoglobin level, 13 (IQR 12–14) g/dl.

### Socio-demographic characteristics of optimal and suboptimal responders

Age, gender and HAART regimen were similar among optimal and suboptimal responders (p value= 0.290, 0.062 and 0.340 respectively. Suboptimal responders had lower CD4 counts at the time of HAART initiation as wells as years after therapy (see Table 1).

**Table 1:** Characteristics of 128 patients with sustained viral suppression after 4 years of antiretroviral therapy at the Infectious Diseases Institute research cohort

**Median CD4 increase ϕ median (range) cells/μl**	**Suboptimal CD4 responders (n=39) 165 (-43-298);]**	**Optimal CD4 reconstitution (n=48) 528 (Range 417-878)**	P value*
Age (yrs) [median (IQR)]	37 (31-42)	35 (31-43)	0.875
Female gender [n (%)]	28 (72)	22 (46)	0.062
Baseline CD4 count cells/μl [median (IQR)]¥	78 (2-117)	115 (97-185)	0.018
<100 cells/μl [n(%)]	26 (67)	7 (15)	0.0015
Current CD4 cells/μl [median (IQR)]	214 (190-282)	474 (438-645)	<0.001
BMI [median (IQR)]	22 (20-25)	26 (22-28)	0.162
Hemoglobin [median (IQR)]	14 (12-15)	14 (13-15)	0.481
Hepatitis B positive [n]	2	1	0.911
First-line HAART regimen∞			
D4T-3TC-NVP/EFZ [n (%)]	12 (31)	27 (56)	
ZDV-3TC-NVP/EFZ [n (%)]	25 (64)	17 (35)	
TDF-3TC-NVP/EFZ [n (%)]	2 (5)	4 (8)	0.340

*Chi square test was used for categorical variables and Kruskal-Wallis test was used for the continuous variables.

ϕThe magnitude of CD4 increase from the baseline counts was grouped into 4 quartiles; ‘Suboptimal responders’ were patients within the lowest quartile and ‘Optimal responders’ were individuals within the highest quartile of CD4 increase.

¥All patients initiated antiretroviral therapy at CD4 200 cells/μl and below.

∞ Study participants were all on their initial first-line regimen given that they had sustained viral suppression

### T-cell proliferation after day 5 of stimulation with SEB, PPD, GagA&D and CMVpp65 antigens

**Figure 3:**
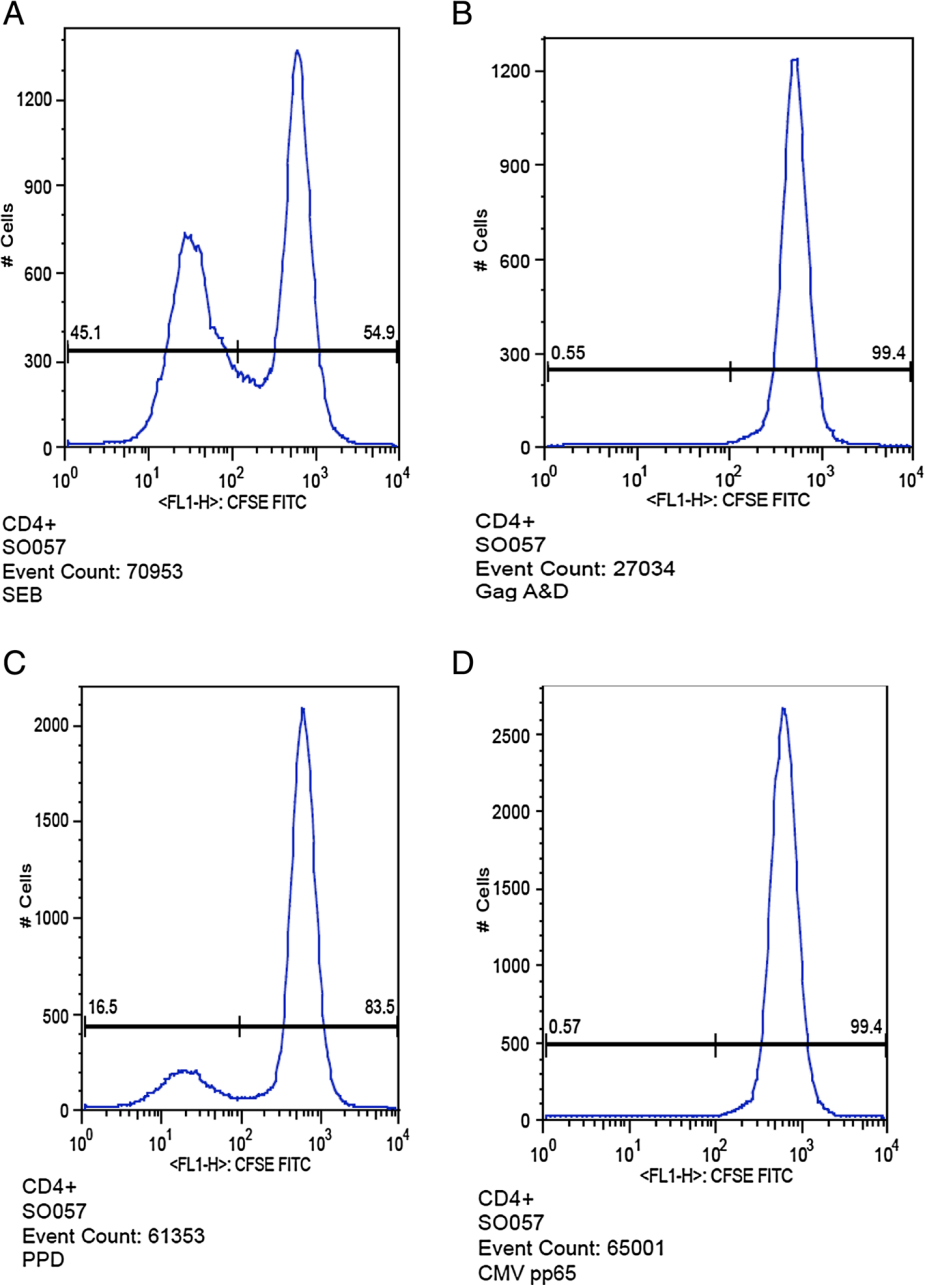
**Typical analysis of T-cell proliferation of peripheral blood mononuclear cells (PBMC) stained labelled with fluorescent dye 5,6-carboxyfluorescein diacetate succinimidyl ester (CFSE); on day 5 of stimulation with SEB, Gag A & D, PPD and CMV antigens.** Panels **A-D** show the typical presentation of T-cell proliferation up stimulation with SEB, Gag A & D, PPD and CMV antigens respectively.

**Figure 4:**
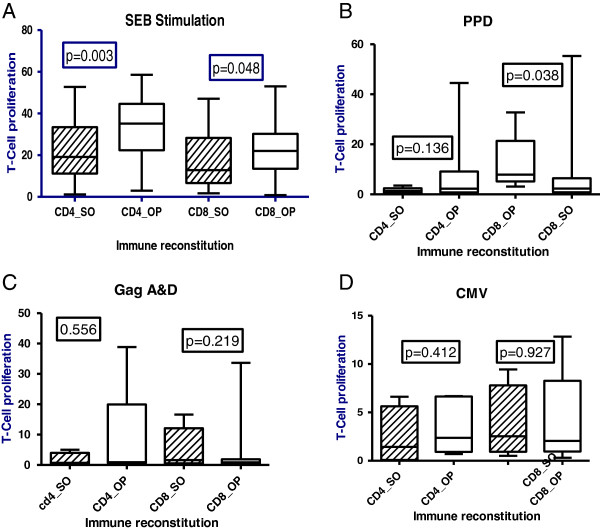
**CD4 and CD8 T-cell proliferation up stimulation by SEB, PPD, GagA&D and CMV antigens respectively; among suboptimal (SO) relative to optimal (OP) immune responders after 4 years of suppressive antiretroviral therapy.** Panel **A** shows lower CD4 and CD8 proliferation among suboptimal relative to optimal responders upon SEB stimulation, Panel **B** shows lower CD8 proliferation among suboptimal relative to optimal responders upon stimulation with tuberculin PPD, Panel **C** shows lower CD4 proliferation upon stimulation with Gag A & D although the difference is not statistically significant and Panel **D** shows lower CD4 proliferation upon stimulation with CMV although the difference is not statistically significant.

**Figure 5:**
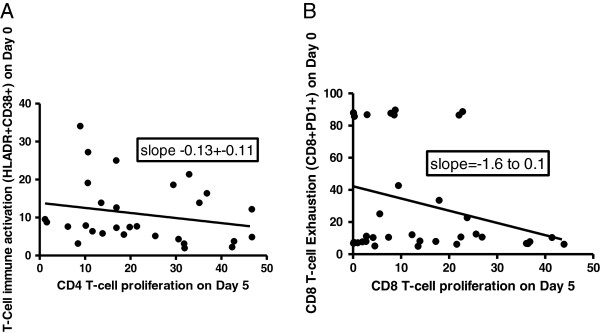
**Correlation of immune activation and exhaustion with T-cell proliferation among suboptimal responders to HAART in a Ugandan cohort.** Panel **A** shows that CD4 T-cell proliferation decreases with increasing levels of immune activation and Panel **B** shows that CD8 T-cell proliferation reduces with increasing levels of exhaustion.

Figure 3 shows our typical analysis of T-cell proliferation of PBMC stained labelled with fluorescent dye 5,6-carboxyfluorescein diacetate succinimidyl ester (CFSE); on day 5 of stimulation with SEB, Gag A & D, PPD and CMV antigens. Upon stimulation with SEB, a super-antigen, T-cell proliferation was lower among suboptimal responders than optimal responders to suppressive HAART; (CD4+ P=0.003 and CD8+P=0.048). Similarly, T-cell proliferation upon stimulation with PPD was lower among suboptimal and optimal responders and the difference was stronger for CD8 T-cells (CD4+; p=0.136 and CD8+; p=0.038); see Figure 4. We found a negative correlation between immune activation and CD4 T-cell proliferation among HAART-treated adults (slope −0.13); as well as a negative correlation between CD8 T-cell exhaustion and proliferation, among suboptimal responders (see Figure 5).

## Discussion

Within the context of sustained viral suppression after 4 years of HAART, we found that suboptimal responders exhibit lower levels of T-cell proliferation than optimal responders. We showed significant differences when PBMCs were stimulated with SEB and PPD. Our results are consistent with previous reports of impaired *in-vitro* HIV-specific CD4 T-cell proliferation among HIV-infected cells although these were not in a setting of suppressive HAART [2,16,17]. In addition to HIV-specific responses, this study analysed T-cell responses to common antigen (SEB, PPD, CMVpp65) to which majority of HIV-infected patients are exposed. Our findings imply that despite sustained viral suppression, the persistently low CD4 T-cell function is likely to predispose suboptimal responders to opportunistic infections and severe *Staphylococcus aureus* infections. This could explain previous reports that HAART-treated individuals with poor CD4 recovery despite viral suppression were at increased risk of opportunistic infections and mortality [12]. Given the low proliferation levels among suboptimal responders, we postulate that suboptimal responders could potentially benefit from longer periods of cotrimoxazole prophylaxis for opportunistic infections as well as additional vaccines of common bacterial infections such as pneumococcal vaccines. With the increasing numbers of individuals on HAART in sub-Saharan Africa, there is clearly need for prospective studies to understand therapeutic interventions for the emerging population of 30-40% suboptimal responders despite viral suppression [12,18] in order to maximise immune recovery during first-line antiretroviral therapy.

The mechanisms of functional impairment of surviving CD4 and CD8 T-cells during chronic HIV infection need to be understood to support the development of targeted interventions. For example, Boasso et al. show that Indoleanine 2,3 dioxyenase (IDO), an immunosuppressive enzyme, plays a role in inhibition of T-cell proliferation and its *in-vitro* inhibition increased CD4 T-cell responses in PBMCs from HIV-infected patients [16]. Immune activation–induced fibrosis of lymphoid tissue in advanced HIV disease is thought to contribute to depletion of naive T-cells and increased apoptosis and subsequently perpetuate cumulative loss of T-cells that may persist despite antiretroviral therapy [19,20]. In addition, recent evidence from mouse studies suggests that lymphoid tissue inducer cells are a relatively newly recognised family of innate lymphoid cells that may have a role in CD4 T-cell responses [21]. Furthermore, HIV-infected dendritic cells produce gp120 that contributes to impaired CD4 T-cell immune responses *in-vivo*; therefore agents that block the gp120-mediated immune suppression could potentially modulate immune responses in HIV-infected individuals [17] and their role in HAART-treated adults may be worth investigation**.** There is clearly a need to understand and explore the role of these mechanisms in CD4 T-cell function recovery within the setting of long-term suppressive HAART. This study contributes to the increasing evidence that monitoring of immune activation levels, viral loads and CD4 function recovery are relevant to compliment the current widely used CD4 count monitoring [3,22] in order to optimise long-term immune recovery within HIV treatment programs in SSA.

We found that CD4 T-cell proliferation decreased with increasing levels of immune activation. Although the exact mechanism is yet to be understood, this data is consistent with our previous report that immune activation and exhaustion were significantly associated with suboptimal CD4 recovery [18]. Similarly, CD8 T-cell proliferation reduced with increasing immune exhaustion. We hypothesize that in addition to low T-cell proliferative ability, the persistently high immune activation and exhaustion levels drive the inflammatory death pathways and subsequently the impairment in CD4 count and functional recovery [23]; however the reverse could also be true. We therefore need to further understand the prevalent T-cell death pathways among suboptimal responders with persistent immune activation.

Our findings are consistent with previous follow-up studies in European and American cohorts that reported failure in long-term restoration of numeric CD4 counts, with the recovery process reaching a plateau after 4–5 years of therapy [5,24-26]. The patients in this study had been on treatment for 4 years which is the likely period for the plateau phenomenon. There is need for follow-up data on specific T-cell responses from this HAART cohort to provide the trends of CD4 count and functional recovery beyond 5 years of therapy. It is also important to note that the patients we studied had initiated HAART in the advanced stages of HIV disease which might be a contributing factor to suboptimal immune recovery [5,25,26] due to the collagen deposition and fibrosis of lymphoid tissue during later stages of HIV infection [4,19,27]. It will be interesting to perform similar assays among patients that initiate HAART at higher CD4 counts (>350 cells as per 2012 national antiretroviral therapy guidelines) or during acute HIV infection, which associated with enhanced likelihood of CD4 count recovery [28]. Due to the limitations of the four color flowcytometry used in this study, we were not able to describe all the T-cell subsets including the memory T-cells of the specific antigens used. This would probably provide more insight into the mechanism of suboptimal immune recovery. For example, previous studies, among HAART-treated individuals with CMV retinitis, showed that memory CMV-specific CD4 and CD8 T-cells expanded several years after HAART initiation [29].

To date, there is no consensus on when and how to treat immunological failure in a setting of viral suppression. Suboptimal responders pose several clinical questions, including questions about the clinical risk associated with persistent immunodeficiency and about possible interventions to optimise clinical and immunological benefits of HAART [12,30]. From a clinical point of view, it is reasonable to hypothesise that adjuvant therapy with anti-immune activation agents [31,32] may improve T-cell proliferation potential among suboptimal responders. Our work presents an interface between clinical care and basic science research. We also add to the evidence that immune modulation could potentially maximize immunological recovery during suppressive antiretroviral therapy.

## Conclusion

CD4 T-cell proliferation was lower among suboptimal than optimal responders, and reduced with increasing levels of CD4 T-cell immune activation markers. T-cell immune activation and exhaustion were associated with poor proliferation among suboptimal responders to HAART despite sustained viral suppression. We recommend prospective studies to understand long-term CD4 numeric and functional recovery among suboptimal responders. In addition, we recommend innovative clinical studies to understand the effect of immune modulants on T-cell function recovery among the emerging populations of suboptimal responders.

## Competing interests

The authors declare that they have no competing interests.

## Authors’ contributions

DN made substantial contribution to the conception, design, data collection, analysis and drafting of the manuscript. IS contributed to the immune assays, data analysis and interpretation. HMK contributed to the conception, design, data interpretation and revision of the manuscript. AK made substantial contribution to the study design and the statistical analysis. RN contributed to the data collection and MRK contributed to the conception, design, data interpretation and revision of the manuscript. All authors read and approved the final manuscript. HC made substantial contribution to the conception, design, immune assays, data analysis, interpretation and revision of the manuscript. All authors read and approved the final manuscript.
